# Pulse Oximetry: 50 Years of Inventions & Discoveries in Biomedical Optics

**DOI:** 10.1117/1.JBO.29.S3.S33301

**Published:** 2025-01-07

**Authors:** Jana M. Kainerstorfer, Kenneth Tichauer, Arjun G. Yodh, Ton van Leeuwen, Brian Pogue

**Affiliations:** a Carnegie Mellon University, Department of Biomedical Engineering, Pittsburgh, Pennsylvania, United States; b Illinois Institute of Technology, Department of Biomedical Engineering, Chicago, Illinois, United States; c University of Pennsylvania, Department of Physics and Astronomy, Philadelphia, Pennsylvania, United States; d Amsterdam University Medical Center, Department of Biomedical Engineering and Physics, Amsterdam, The Netherlands; e University of Wisconsin–Madison, College of Engineering, Department of Medical Physics, Madison, Wisconsin, United States

## Abstract

The editorial introduces the JBO Special Issue on Pulse Oximetry.

In 1974, a Japanese bioengineer named Takuo Aoyagi, working for the Nihon Kohden Corp, developed a working prototype of an optical measurement system that sensed blood pulsation by detection of the temporal variation of light transmission through tissue.^1^^,^^2^ These changes showed that macroscopic measurements of diffuse light could directly sample variation in light absorption caused by dilation and constriction of arterial vessels at the heart rate frequency. Arguably, this electrical engineering advance would become perhaps the most impactful development in deep tissue biomedical optics. The discovery found wider recognition through the work of John Severinghaus,^3^ in the late-1980s, when functional pulse oximeters became commercially realized. All of this progress can be traced to a core discovery that diffuse light transmission through tissue carried temporal signatures with useful information from the microscopic blood vessel network.

The idea that spectroscopic measurement of these temporal signatures could be translated to quantitatively measure arterial blood oxygen saturation was revolutionary. Today, pulse oximeters are ubiquitous in medicine, and the principles discovered in early pulse oximetry have ballooned to inspire many subsequent discoveries. The applications of imaging and sensing with diffuse light are extensive, as are the applications of related temporal and spectroscopic instrumentation and algorithms. In 2024, we celebrated the 50^th^ anniversary of this discovery by a dedicated electrical engineer. The discovery eventually changed mainstream medical care, saving countless lives and transforming our ideas about what is possible with optical measurement through tissue.

This JBO special issue features several contemporary topics in the field of tissue and pulse oximetry with an emphasis on the effects of skin melanin content on oxygenation estimation accuracy, explored through simulation and clinical studies. Additional topics include emerging developments of more realistic solid phantoms for evaluating tissue oximetry technologies, the use of artificial intelligence to improve tissue oxygenation estimation, and the presentation of technological methods to improve dynamic optical imaging approaches for brain oxygenation and tissue perfusion monitoring.

A comprehensive review on the history, status, and outlook of pulse oximetry was contributed by Quaresima, Ferrari, and Scholkmann,^4^ with an emphasis on the origins of tissue oximetry measurement. Providing further context to the underpinnings of pulse oximetry, Blaney, Sassaroli, and Fantini^5^ offered a technical perspective on the relationship between arterial oxygen saturation of hemoglobin, and the ratio-of-ratios approach used in standard pulse oximetry to estimate the arterial oxygen saturation of hemoglobin.

With the recent COVID-19 pandemic magnifying the impact of the reduced accuracy of current pulse oximetry designs for quantifying arterial oxygenation in individuals with darker skin, several articles focused on elucidating the mechanisms of the effect of melanin on optical oximetry and proposed solutions to correct for the bias. Research papers from Al-Halawani, Qassem, and Kyriacou;^6^ Narayanaswamy et al.,^7^ and Blaney et al.^8^ all presented Monte Carlo simulation results to uncover the causes of inaccurate blood oxygenation estimation with pulse oximetry in people with dark skin; a research paper from Roy et al.^9^ explored the effect of melanin on frequency-domain near-infrared spectroscopy measurements; and research papers from Bhusal et al.^10^ and Ma et al.^11^ proposed new methods of solid optical phantom construction that could be used for more realistic testing of pulse oximeter performance with respect to variation in skin pigmentation.

Considering the benefits of machine learning and artificial intelligence for nearly all aspects of our lives, three articles investigated ways in which artificial intelligence can help to improve methods of tissue oxygenation estimation. Gröhl et al.^12^ introduced a recurrent neural network architecture for spectral unmixing to improve the accuracy of blood oxygenation estimation using multi-spectral photoacoustic data, while Larsson et al.^13^ and Liao et al.^14^ developed frameworks to train artificial neural networks that estimate blood oxygen saturation from non-contact multispectral optical data.

Finally, Kamar et al.^15^ assessed the “Kernel Flow,” a high-density, time-domain cerebral oxygenation measurement system with advantages in distinguishing signals from the brain and scalp,^16^ while Tang et al.^17^ compared measurement of the arterial input function of indocyanine green using pulse dye densitometry—a method closely linked to pulse oximetry—against no arterial input function or population-based arterial input function assumption for quantifying tissue perfusion in dynamic fluorescence imaging studies.

Despite the long history and nearly ubiquitous use of pulse oximetry throughout healthcare worldwide, there are reinvigorated efforts to improve the technology—particularly with respect to the effect that darker skin has on the accuracy of devices—and adopt its founding principles to a greater number of protocols and applications. While this JBO special issue commemorates one of the greatest achievements in biomedical optics thus far, on the 50^th^ anniversary of the development of the first pulse oximeter, we intend that the issue also highlights the ongoing efforts and further need for continued development of the technology.
